# On the Causes of Death after Amputation

**Published:** 1859-10

**Authors:** 

**Affiliations:** Assistant Surgeon to Guy’s Hospital


*On the Causes of Death after Amputation.* By Mr. Bryant, Assistant Surgeon to Guy's Hospital. (*Proc. of the Roy. Med. and Chir. Soc., Feb.* 22, 1859).

This paper is based upon an analysis of 300 cases of amputation, collected from the records of Guy's Hospital.

The author has divided them into four classes; and although he has not thought it necessary to alter the ordinary division of traumatic amputations into primary and secondary, he has made some change in the division of the other forms; for it became evident in the analysis, that the classing together of such cases as amputations for talipes, tumors, elephantiasis, deformity, and others of a like character, with those of diseases of the joints, a wrong result must ensue; and practically, this was found to be the case. He therefore divides these cases into pathological amputations and amputations of expediency, choosing the latter term as more accurately expressing the reason for the operation, as limbs are removed for tumor, talipes, elephantiasis and deformity more from expediency than necessity, and he therefore suggests the use of such term until a better be proposed.

*General Conclusions upon the Causes of Death in Amputations generally.*—1. That 25 per cent. are fatal; 30 per cent. of the lower extremity, 10 per cent. of the upper. 2. That pyæmia is the cause of death in 42 per cent. of the fatal cases, and 10 per cent. of the whole number amputated. 3. That exhaustion is the cause of death in 33 per cent. of the fatal cases, and in 8 per cent. of the whole number amputated. 4. That the following causes of death are fatal in the annexed proportions:

| | Of Fatal Cases. | Of whole number. |
|---|---|---|
| Secondary hemorrhage, . . | 7 per cent. | or 1.66 per cent. |
| Thoracic complications, . . | 5.6 " | or 1.33 " |
| Cerebral, . . . . . . . . . | 3 " | or .66 " |
| Abdominal, . . . . . . . | 1.4 " | or .33 " |
| Renal, . . . . . . . . . . | 3 " | or .66 " |
| Hectic, . . . . . . . . . . | 3 " | or .66 " |
| Traumatic, . . . . . . . . | 7 " | or 1.66 " |

*Pathological Amputations.*—1. That pathological are by far the most successful amputations, 12.5 per cent. proving fatal. Such amputations of the upper extremity are generally followed by success. Of the lower extremity, 15 per cent. terminate fatally. 2. That pyæmia is the chief cause of death, proving fatal in 43 per cent. of the fatal cases and in 5.4 per cent. of all pathological amputations; and when fatal, as a rule, it causes death within fourteen days of the operation. 3. That exhaustion, either from the shock of the accident or of the operation, from hemorrhage, or all three causes combined, is the cause in 33 per cent. of the fatal cases, or 4 per cent. of all amputations. 4. That secondary hemorrhage is the fatal cause in only 9 per cent. of the fatal cases, and in 1.4 per cent. of all amputations.

*Amputations of expediency.*—1. That 30 per cent. are fatal; but as amputations of the upper extremity are, as a rule, successful, the per centage of this operation upon the lower is much increased, 40 per cent. proving fatal. 2. That pyæmia is the chief cause of death, proving fatal in 60 per cent. of the fatal cases, and in 18 per cent. of all such amputations; and when fatal, as a rule, death takes place within fourteen days of the operation. 3. That death from exhaustion occurs in but 10 per cent. of the fatal cases; and that some thoracic or renal complication, or carcinomatous infiltration, are fatal causes in the same proportion.

*Primary Amputations.*—1. That 43 per cent. are fatal; 60 per cent. of the lower extremity, and 30 per cent. of the upper. 2. That primary amputations are more successful than secondary. 3. That pyæmia is the cause of death in 43 per cent. of the fatal cases and in 16 per cent. of the whole number, and that, when fatal, the symptoms appear, as a rule, between the seventh and fourteenth days after the operation, and cause death in the third or fourth week, and not during the first two weeks, as in pathological amputations and those of expediency. 4. That exhaustion is the cause of death in 32 per cent. of the fatal cases, and in 12 per cent. of the whole number. 5. That traumatic complications prove fatal in 15 per cent. of the fatal cases, and secondary hemorrhage, cerebral or thoracic complications, about 7 per cent. each; renal disease proving a cause of death in 3.5 per cent.

*Secondary Amputations.*—1. That 50 per cent. are fatal; 68 of the lower extremity, and 12.5 per cent. of the upper. 2. That secondary amputations are more fatal than primary, by about 8 per cent. 3. That exhaustion is the chief cause of death, proving the cause in 60 per cent. of the fatal cases.

4. That pyæmia is the cause in 25 per cent. of the fatal cases; secondary hemorrhage and hectic in the remaining 15 per cent.

*Conclusions upon Pyœmia as a Cause of Death.*—1. That it is the cause of death in 42 per cent. of all fatal cases of amputations, and in 10 per cent. of all amputations. 2. That it is the cause of death in the different forms of amputation in the following order:—1. In 70 per cent. of all fatal amputations of expediency. 2. In 43 per cent. of all fatal primary amputations. 3. In 43 per cent. of all fatal pathological amputations. 4. In 25 per cent. of all fatal secondary amputations, and that, in amputations of expediency, it is the most frequent cause, and in secondary amputations, the least. 5. That in amputations for acute suppuration of the knee-joint, whether the result of an abscess discharging into the joint or otherwise, pyæmia is a more frequent cause of death, than in amputations for chronic disease. 6. That it is the general cause of death for talipes, elephantiasis and tumors. 7. That in primary amputations, and in amputations of expediency of the leg, it is a more frequent cause of death, than in the same operations upon the thigh. 8. That, upon the whole, pyæmia appears to be a more frequent cause of death in amputations through limbs the tissues of which are in a normal condition, and where a large surface of healthy bone is exposed. 9. That in pathological amputations, and in amputations of expediency, pyæmia, as a rule, proves fatal within fourteen days; but after traumatic operations, the period of death is about the twenty-fifth or twenty-sixth day.

*General Conclusions upon Amputations of the Thigh.*—1. That 27 per cent. are fatal. Pathological amputations, 18 per cent.; amputations of expediency, 31 per cent.; primary amputations, 60 per cent.; secondary, 75 per cent. 2. That in amputation of the thigh for chronic disease of the knee-joint, about 16 per cent. are fatal, or 1 case in 7. 3. That amputations of the thigh for acute suppuration in the joint are generally fatal, and that pyæmia is the chief cause of death in these cases. 4. That exhaustion and pyæmia are causes of death in equal proportions; or in about 40 per cent. of the fatal cases, and in 10 per cent. of all amputations of the thigh. 5. That exhaustion is most fatal in primary amputations, and the least so in amputations of expediency. 6. That pyæmia is most fatal in amputations of expediency, and the least so in primary. 7. That primary amputations are for the most part fatal from exhaustion; 35 per cent. of the cases sinking from this cause, 15 per cent. from pyæmia, and secondary hemorrhage and traumatic complications 5 per cent. each. 8. That exhaustion, pyæmia and hectic are equally fatal causes in secondary amputations, proving fatal in 25 per cent. each.

*Amputations of the Leg.*—1. That 37 per cent. are fatal. Pathological amputations, 7.7 per cent.; amputations of expediency, 66.6 per cent.; primary amputations, 62.5 per cent.; secondary amputations, 66.6 per cent. 2. That amputations of the leg are 10 per cent. more fatal than of the thigh; the amputations of expediency and traumatic amputations being more fatal, and the latter more frequent. 3. That amputations of expediency of the leg are generally fatal—being twice as fatal as those of the thigh: that pyæmia is the chief cause of death in 75 per cent. of the fatal cases, and in 50 per cent. of all such amputations. 4. That in primary amputations pyæmia is the death in half the fatal cases, or in 32 per cent. of all such operations; exhaustion and visceral complications about 8 per cent. each. 5. That comparing primary amputations of the thigh and leg together they are equally fatal, but that pyæmia is twice as fatal in amputations of the leg as in amputations of the thigh. 6. That half the cases of secondary amputations die from exhaustion; pyæmia and secondary hemorrhage being fatal in 8 per cent. each. 7. That taking all amputations of the leg together, 42 per cent. of the fatal cases die from pyæmia and 32 per cent. from exhaustion.

*Amputations of the Upper Extremity.*—1. That 10 per cent. are fatal. 2. That pathological and those of expediency are, as a rule, successful. 3. That about 20 per cent. of traumatic amputations are fatal; 22 per cent. of the arm and 16 per cent. of the forearm. 4. That one-third of these fatal cases die from pyæmia, one-third from some traumatic complication, and the remaining third from secondary hemorrhage or visceral disease. —*Ranking's Abstract.*

